# Serum MicroRNAs as Biomarkers in Hepatitis C: Preliminary Evidence of a MicroRNA Panel for the Diagnosis of Hepatocellular Carcinoma

**DOI:** 10.3390/ijms20040864

**Published:** 2019-02-17

**Authors:** Anna Weis, Louise Marquart, Diego A. Calvopina, Berit Genz, Grant A. Ramm, Richard Skoien

**Affiliations:** 1 Hepatic Fibrosis Group, QIMR Berghofer Medical Research Institute, 300 Herston Rd, Herston, QLD 4006, Australia; anna.weis1@uqconnect.edu.au (A.W.); Diego.Calvopina@qimrberghofer.edu.au (D.A.C.); Berit.Genz@qimrberghofer.edu.au (B.G.); Richard.Skoien@health.qld.gov.au (R.S.); 2 Faculty of Medicine, University of Queensland, Herston Road, Herston, QLD 4006, Australia; 3 QIMR Berghofer Statistics Unit, QIMR Berghofer Medical Research Institute, 300 Herston Rd, Herston, QLD 4006, Australia; Louise.Marquart@qimrberghofer.edu.au; 4 Department of Gastroenterology and Hepatology, Royal Brisbane and Women’s Hospital, Bowen Bridge Rd & Butterfield St, Herston, QLD 4029, Australia

**Keywords:** serum miRNA, hepatocellular carcinoma, cirrhosis, non-invasive biomarker, hepatitis C

## Abstract

Early diagnosis of cirrhosis and hepatocellular carcinoma (HCC) due to chronic Hepatitis C (CHC) remain clinical priorities. In this pilot study, we assessed serum microRNA (miRNA) expression to distinguish cirrhosis and HCC, alone and in combination with the aminotransferase-to-platelet ratio (APRI), Fibrosis 4 (FIB-4), and alpha-fetoprotein (AFP). Sixty CHC patients were subdivided into 3 cohorts: Mild disease (fibrosis stage F0-2; *n* = 20); cirrhosis (*n* = 20); and cirrhosis with HCC (*n* = 20). Circulating miRNA signatures were determined using a liver-specific real-time quantitative reverse transcription PCR (qRT-PCR) microarray assessing 372 miRNAs simultaneously. Differentially-expressed miRNA candidates were independently validated using qRT-PCR. Serum miRNA-409-3p was increased in cirrhosis versus mild disease. In HCC versus cirrhosis, miRNA-486-5p was increased, whereas miRNA-122-5p and miRNA-151a-5p were decreased. A logistic regression model-generated panel, consisting of miRNA-122-5p + miRNA-409-3p, distinguished cirrhosis from mild disease (area under the curve, AUC = 0.80; sensitivity = 85%, specificity = 70%; *p* < 0.001). When combined with FIB-4 or APRI, performance was improved with AUC = 0.89 (*p* < 0.001) and 0.87 (*p* < 0.001), respectively. A panel consisting of miRNA-122-5p + miRNA-486-5p + miRNA-142-3p distinguished HCC from cirrhosis (AUC = 0.94; sensitivity = 80%, specificity = 95%; *p* < 0.001), outperforming AFP (AUC = 0.64, *p* = 0.065). Serum miRNAs are differentially expressed across the spectrum of disease severity in CHC. MicroRNAs have great potential as diagnostic biomarkers in CHC, particularly in HCC where they outperform the only currently-used biomarker, AFP.

## 1. Introduction

Chronic Hepatitis C (CHC) is a major risk factor for the development of cirrhosis and subsequent hepatocellular carcinoma (HCC), a cancer whose incidence and mortality are increasing [1]. The impact of new, highly efficacious antiviral therapies [2] on morbidity and mortality largely depends on improved identification of cirrhosis due to CHC and its complications, including HCC. Despite treatment, the burden of CHC-related HCC in most countries is increasing and is expected to peak in the next decade [3]. Furthermore, whilst achieving a sustained virologic response (SVR) has been shown to improve liver function in cirrhotic patients, the risk of complications, such as HCC, remains [4]. There remain clinical imperatives to accurately identify patients with cirrhosis and improve diagnostic tests for CHC-related HCC.

The gold standard for the identification of cirrhosis for the purposes of HCC surveillance is liver biopsy, but this is invasive, prone to sampling error, and does not reflect the clinical spectrum of cirrhosis and is therefore now rarely used. Non-invasive tools, such as liver stiffness assessment (e.g., transient elastography), have been embraced to stage fibrosis in CHC patients, but a clinically-significant “indeterminate” range affecting the test accuracy exists [5]. Non-invasive biomarker panels, such as the aspartate aminotransferase to platelet ratio (APRI) [6] and Fibrosis-4 (FIB-4) [7] scores, provide good sensitivity and specificity within defined cut-offs, but values falling short of these specific thresholds lack diagnostic accuracy. Access to non-invasive tools is also sometimes limited to specialist treatment, pathology, or imaging centres.

The only currently-accepted HCC tumour biomarker, alpha-fetoprotein (AFP), generally has suboptimal sensitivity and specificity for surveillance and diagnosis in most cases [8]. Routine HCC surveillance comprises 6-monthly ultrasounds, with dynamic scanning (CT/MRI) used to further characterise suspicious lesions. For a proportion of tumours, however, diagnosis is necessarily delayed until interval growth can be demonstrated, limiting early access to definitive treatment. Indeed, although the sensitivity and specificity of ultrasound-based diagnostic strategies exceed 90% for the detection of HCC across all stages, its sensitivity is only 63% for early HCC [8].

Novel biomarkers, such as microRNAs (miRNAs), are being investigated for their diagnostic potential in viral hepatitis, hepatic fibrosis, and HCC. miRNAs are small non-coding RNAs involved in the regulation of gene expression at a post-transcriptional level. The stability of miRNAs in the circulation, as well as both healthy and diseased tissue, make them ideal potential biomarkers [9]. Preliminary studies of miRNAs in CHC-related HCC have identified promising candidates [10,11], with the development of miRNA panels now being explored to improve diagnostic accuracy.

In this pilot study, we sought to identify differentially-expressed serum miRNAs in CHC patients with and without cirrhosis, as well as those with CHC-related HCC. We also sought to develop miRNA panels of the most promising diagnostic candidates, alone or in combination with APRI, FIB-4, or AFP, to explore their potential as novel non-invasive diagnostic tests.

## 2. Results

### 2.1. Patient Characteristics

The patient demographics and clinical characteristics of the CHC patient cohort are summarised in Table S2. All patients with mild disease (F0-2) and cirrhosis (F4) were followed for a mean of 16.7 and 21.2 months from sample collection, respectively, without any evidence of clinical decompensation or HCC development. There were no significant differences in CHC genotypes or treatment history between the three patient cohorts (*p* > 0.05).

### 2.2. Differential miRNA Expression in Chronic Hepatitis C (CHC)

**Figure 1:**
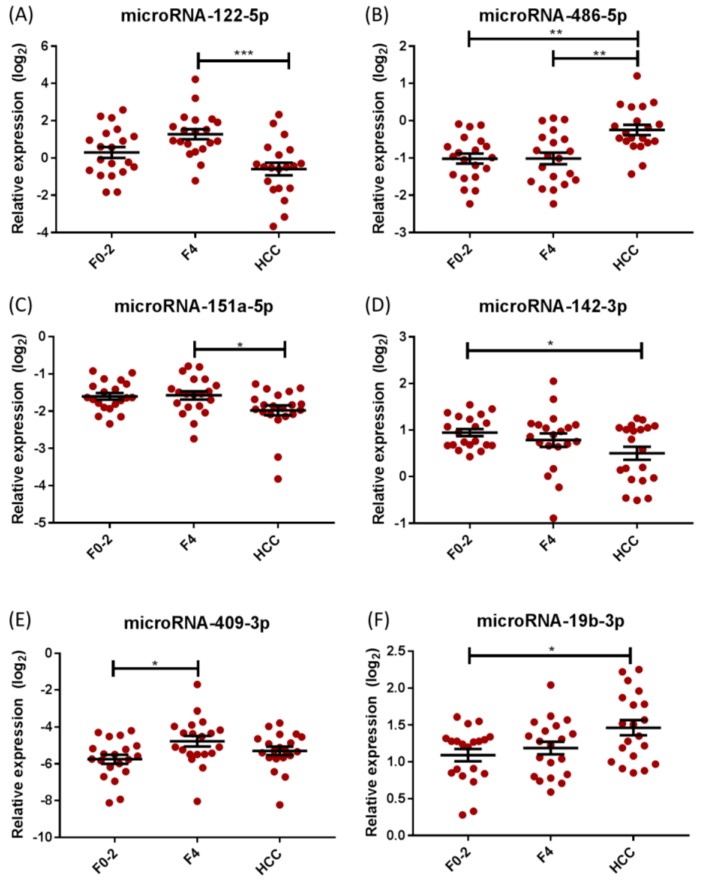
Association between circulating miRNA levels and severity of hepatic fibrosis and HCC in CHC. (**A**) miRNA-122-5p, (**C**) miRNA-151a-5p, and (**D**) miRNA-142-3p show a decreased expression in HCC, compared to cirrhosis, whereas (**B**) miRNA-486-5p shows an increased expression in HCC, compared to cirrhosis. (**E**) miRNA-409-3p shows an increased expression in F4, when compared to mild disease. (**F**) miRNA-19b-3p shows an increased expression in HCC compared to mild disease. Lines represent mean expression levels (±SEM). * *p* < 0.05, ** *p* < 0.01, *** *p* < 0.001. (Abbreviations: CHC = chronic Hepatitis C; F0-2 = mild disease; F4 = cirrhosis; HCC = hepatocellular carcinoma; miRNA = microRNA; SEM = standard error of the mean).

Seven candidate miRNAs with 2-fold or more differential expression in F0-2 vs. F4 vs. HCC on the microarray were identified for further validation: miRNA-19b-3p, miRNA-22-3p, miRNA-122-5p, miRNA-142-3p, miRNA-151a-5p, miRNA-409-3p, and miRNA-486-5p. Differential expression levels on qRT-PCR of the selected miRNA candidates are presented in Figure 1. miRNA-409-3p was significantly upregulated in cirrhosis compared with mild disease (*p* = 0.023; Table 1 and Figure 1).

**Figure 2:**
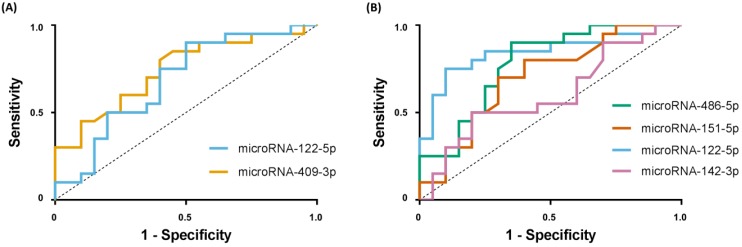
ROC curves for individual serum miRNAs to identify cirrhosis or HCC in chronic hepatitis C (**A**). ROC curve performance of miRNA-122-5p with AUC of 0.69 (95% CI 0.51–0.86, *p* = 0.023) and miRNA-409-3p with AUC of 0.74 (95% CI 0.58–0.90, *p* = 0.004) to identify cirrhosis when compared to mild disease. (**B**) ROC curve performance of selected miRNAs to identify HCC when compared to cirrhosis as follows: miRNA-486-5p, AUC of 0.78 (95% CI 0.63–0.93, *p* < 0.001); miRNA-151a-5p, AUC of 0.70 (95% CI 0.54–0.87, *p* = 0.014); miRNA-122-5p, AUC of 0.85 (95% CI 0.72–0.97, *p* < 0.001); miRNA-142-3p, AUC of 0.60 (95% CI 0.42–0.78, *p* = 0.140). (Abbreviations: AUC = area under the curve; HCC = hepatocellular carcinoma; miRNA = microRNA; ROC = receiver operating characteristic; 95% CI = 95% confidence interval).

In HCC, serum levels of miRNA-122-5p and miRNA-151a-5p were significantly decreased (*p* < 0.001 and *p* = 0.039, respectively), whereas expression of miRNA-486-5p was significantly increased in HCC patients (*p* = 0.001), compared with F4 patients (Table 1, Figure 1). Expression of miRNA-142-3p was significantly decreased, while miRNA-19b-3p was significantly increased, in HCC compared with mild disease (*p* = 0.039 and *p* = 0.015, respectively; Table 1, Figure 1). Significant differential expression of miRNA-22-3p was not confirmed on qRT-PCR, with only a trend towards increased expression in cirrhosis vs. mild disease (*p* = 0.056). Using receiver operating characteristic (ROC) curves, area under the curve (AUC) analysis was performed to assess the potential of individual candidate miRNAs to discriminate cirrhosis (from mild disease) and HCC (from cirrhosis alone), with fair performance only (AUCs 0.60–0.85; Figure 2).

### 2.3. Discriminating Cirrhosis and Hepatocellular Carcinoma (HCC) Using Serum miRNA Panels

**Figure 3:**
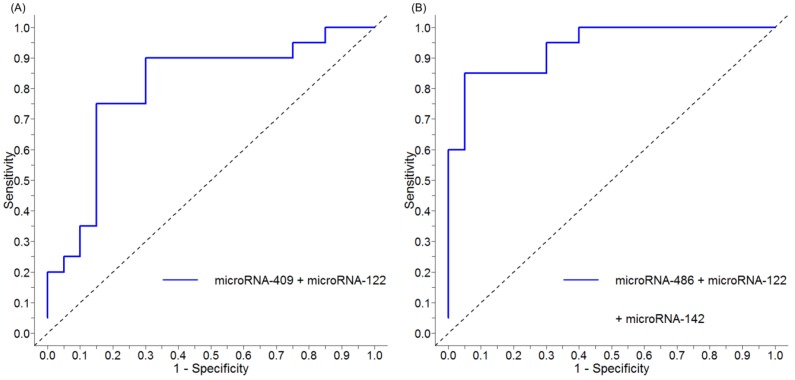
ROC curve of serum miRNA panels to identify cirrhosis or HCC in chronic Hepatitis C (**A**) miRNA panel (miRNA-122-5p and miRNA-409-3p) performance to identify cirrhosis, compared to mild disease, with AUC of 0.80 (95% CI 0.66–0.95, *p* < 0.001). (**B**) miRNA panel to identify HCC, compared to cirrhosis, using miRNA-122-5p, miRNA-486-5p, and miRNA-142-3p with AUC of 0.94 (95% CI 0.87–1.00, *p* < 0.001). (Abbreviations: AUC = area under the curve; F0-2 = mild disease; F4 = cirrhosis; HCC = hepatocellular carcinoma; miRNA = microRNA; ROC = receiver operating characteristic; 95% CI = 95% confidence interval).

We used stepwise logistic regression, using forward selection and backward elimination, to develop serum miRNA panels that could distinguish cirrhosis or HCC (from mild disease and cirrhosis, respectively). A panel comprising miRNA-122-5p and miRNA-409-3p demonstrated optimal differential diagnostic performance for cirrhosis (vs. F0-2) with an AUC of 0.80 (95% CI 0.66–0.95, *p* < 0.001), sensitivity of 85%, specificity of 70%, negative predictive value (NPV) of 82%, positive predictive value (PPV) of 74%, and overall accuracy of 78% (Figure 3A). For HCC (vs. F4), an optimal panel comprising miRNA-122-5p, miRNA-486-5p, and miRNA-142-3p demonstrated excellent diagnostic performance with an AUC of 0.94 (95% CI 0.87–1.00, *p* < 0.001), sensitivity of 80%, specificity of 95%, NPV of 94%, PPV of 83%, and overall accuracy of 88% (Figure 3B).

### 2.4. Cross Validation of Serum miRNA Panels

**Figure 4:**
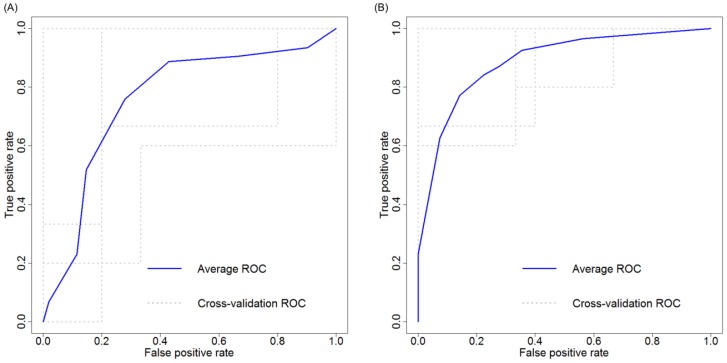
K-fold (5-fold) cross validation ROC curve of serum miRNA panels to identify cirrhosis or HCC in chronic Hepatitis C (**A**) miRNA panel (miRNA-122-5p and miRNA-409-3p) 5-fold cross validation performance to identify cirrhosis, compared to mild disease, demonstrates an AUC of 0.79 (95% CI 0.65–0.93). (**B**) miRNA panel to identify HCC, compared to cirrhosis, using miRNA-122-5p, miRNA-486-5p, and miRNA-142-3p, demonstrates an AUC of 0.91 (95% CI 0.81–1.0). (Abbreviations: AUC = area under the curve; F0-2 = mild disease; F4 = cirrhosis; HCC = hepatocellular carcinoma; miRNA = microRNA; ROC = receiver operating characteristic; 95% CI = 95% confidence interval).

To further assess the robustness of the miRNA panel diagnostic performance, k-fold cross validation was performed using 5-fold cross-validation. Our cirrhosis miRNA panel, combining miRNA-409-3p and miRNA-122-5p, resulted in an AUC of 0.79 (95% CI 0.65–0.93), with a sensitivity of 85%, specificity of 65%, NPV of 76%, PPV of 75%, and overall accuracy of 78% (Figure 4A). Our HCC miRNA panel, combining miRNA-122-5p, miRNA-486-5p, and miRNA-142-3p, resulted in an AUC of 0.91 (95% CI 0.81–1.0), sensitivity of 81%, specificity of 87%, NPV of 80%, PPV of 91%, and overall accuracy of 85% (Figure 4B).

### 2.5. Comparative Performance of the Aspartate Aminotransferase-to-Platelet Ratio (APRI) and Fibrosis 4 (FIB-4) ± Serum miRNA Panel to Discriminate Cirrhosis

**Figure 5:**
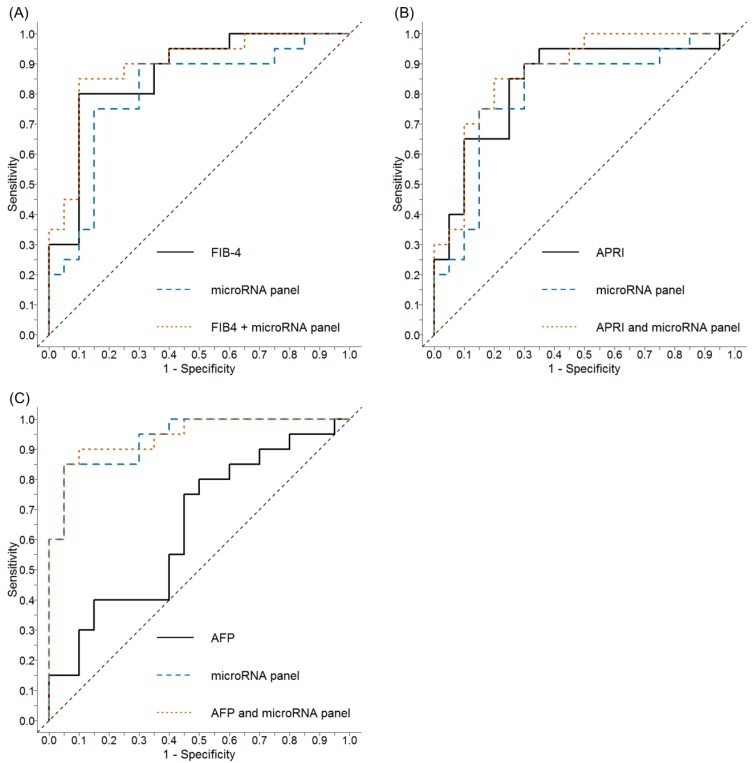
ROC curves to identify cirrhosis or HCC using FIB-4 or APRI alone, or in combination with circulating miRNA panels in chronic Hepatitis C. (**A**) In distinguishing cirrhosis, the performance of FIB-4 (AUC = 0.87, 95% CI 0.75–0.98, *p* < 0.001; using a cut-off of >1.45 to exclude advanced fibrosis) was enhanced in combination with the cirrhosis miRNA panel described in Figure 3A (AUC = 0.89, 95% CI 0.79–0.99, *p* < 0.001); (**B**) in distinguishing cirrhosis, the performance of APRI (AUC = 0.84, 95% CI 0.71–0.97, *p* < 0.001; using a cut-off of >1.0 to exclude cirrhosis) was enhanced in combination with the cirrhosis miRNA panel described in Figure 3A (AUC =0.87, 95% CI 0.76–0.98, *p* < 0.001); (**C**) AFP alone demonstrated poor diagnostic utility in the detection of HCC (AUC = 0.64, 95% CI 0.47–0.82, *p* = 0.065; using a cut-off of >20 to diagnose HCC) and when used in combination with the HCC miRNA panel described in Figure 3B there was no improvement in the HCC miRNA panel’s ability to detect HCC (AUC = 0.94, 95% CI 0.88–1.00, *p* < 0.001). (Abbreviations: APRI = aspartate aminotransferase to platelet ratio; AFP = alpha-fetoprotein; AUC = area under the curve; FIB-4 = Fibrosis-4 biomarker panel; HCC = hepatocellular carcinoma; miRNA = microRNA; ROC = receiver operating characteristic; 95% CI = 95% confidence interval).

The diagnostic performance of the biochemical indices, FIB-4 and APRI, to discriminate cirrhosis (F4) from mild disease (F0-2) in our cohort was also assessed. Using the currently accepted FIB-4 cut-off of 1.45 (to exclude advanced fibrosis) resulted in an AUC of 0.87 (95% CI 0.75–0.98, *p* < 0.001) with a sensitivity of 95%, specificity of 50%, NPV of 92%, PPV of 70%, and overall accuracy of 78% (Figure 5A). Applying the commonly accepted APRI cut-off of 1.0 (to exclude cirrhosis) resulted in an AUC of 0.84 (95% CI 0.71–0.97, *p* < 0.001), with a sensitivity of 80%, specificity of 75%, NPV of 79%, PPV of 76%, and overall accuracy of 78% (Figure 5B). Combining these scores with our cirrhosis miRNA panel marginally improved the diagnostic performance with AUCs of 0.87 and 0.89 for APRI and FIB-4, respectively.

### 2.6. Comparative Performance of Alpha-Fetoprotein (AFP) ± Serum miRNA Panel to Discriminate HCC

In this cohort, the currently accepted tumour marker, AFP, was a poor discriminator of HCC with an AUC of 0.64 (95% CI 0.47–0.82, *p* = 0.065). Using a commonly accepted AFP cut-off of >20 µg/L to identify HCC produced a sensitivity of 25%, specificity of 90%, NPV of 55%, PPV of 71%, and overall accuracy of 58%. Combining AFP with our HCC miRNA panel did not improve the diagnostic performance with an AUC of 0.94 (95% CI 0.88–1.00, *p* < 0.001; Figure 5C).

## 3. Discussion

Despite the availability of new, highly-efficacious treatments, CHC remains a huge public health burden with millions of infected patients affected by cirrhosis worldwide [3]. Distinguishing cirrhosis from mild disease remains essential as such patients warrant ongoing surveillance for complications, such as HCC, even after SVR. The invasive nature of the gold standard liver biopsy and the absence of highly accurate non-invasive biomarkers remain important challenges in routine patient care [12]. Although miRNAs demonstrate potential as biomarkers, with the identification of several candidate miRNAs in many diseases (including HCC), optimal diagnostic panels have not been developed.

In this pilot study, we used serum samples from 60 well-characterised CHC patients across the spectrum of disease to systematically identify the most promising potential miRNA biomarkers. During the discovery phase, differentially-expressed miRNAs were identified via liver-specific microarray. The most promising candidates were then validated using independent qRT-PCR and then optimised as miRNA panels to test their diagnostic accuracy compared with currently-accepted non-invasive clinical tools. Our data strengthen the case for further exploration of serum miRNAs as clinically-useful biomarkers in cirrhosis and HCC.

The circulating serum level of miRNA-409-3p was significantly increased in cirrhotic patients (F4) compared to those with only mild disease (F0-2). There is little in the literature describing the role of miRNA-409-3p in fibrosis progression in liver disease, although increased plasma expression levels correlated with markers of liver injury (gamma-glutamyl-transferase, alkaline phosphatase) in a murine neoplastic model of hereditary tyrosinemia type 1 [13]. As a cancer biomarker, studies using in vitro models of prostate cancer have reported increased miRNA-409-3p levels [14]. Interestingly, this was in association with epithelial to mesenchymal transition, a purported mechanism of fibrosis progression in liver disease. Reduced expression of miRNA-409-3p in other cancers, such as breast cancer [15], suggests a tissue-specific role for miRNA-409-3p.

miRNA-122-5p is the most abundant miRNA expressed in the liver [16]. In CHC, circulating miRNA-122-5p levels have been shown to correlate with serum ALT and AST levels and tissue necrosis and inflammation [11,17]. In the current study, we demonstrated that circulating serum levels of miRNA-122-5p were significantly decreased in CHC patients with HCC compared with cirrhosis alone. A previous study reported no significant differences in serum miRNA-122-5p levels in HCC patients [18], but this study included patients with multiple disease aetiologies rather than CHC patients alone. Conversely, increased HCC tissue expression levels have been described for miRNA-122-5p, including CHC-related HCC [19,20]. Differential peripheral versus tissue expression is commonly-described with miRNAs in cancer, highlighting their potential application as clinical biomarkers, although specific expression profiles are yet to be definitively established. For example, reduced tissue expression in HCC has been shown to correlate with poor prognosis and metastasis [19], while poorer overall survival has also been described in HCC patients with lower levels of circulating miRNA-122-5p [18]. While the precise mechanisms involved require further investigation, these data clearly suggest the clinical importance of miRNA-122-5p in HCC and the current study provides consistent evidence that miRNA-122-5p may have utility as a non-invasive serum biomarker in HCC.

In the current study, circulating miRNA-486-5p was the only miRNA with an increased peripheral expression in CHC-related HCC compared with cirrhosis alone. Higher levels of circulating miRNA-486-5p have been previously described in patients with HCC compared with healthy controls, although the aetiology of HCC and cirrhosis status was not reported [21]. Higher serum expression of miRNA-486-5p has been associated with longer recurrence-free survival in HCC patients [22], while reduced expression in HCC tissue compared with adjacent non-tumorous tissue has been reported in multiple chronic liver disease aetiologies, including CHC-related HCC [23,24]. Expression levels also appear to correlate with earlier HCC recurrence following resection [23]. Downstream targets of miRNA-486-5p include Phosphoinositide-3-Kinase Regulatory Subunit 1 (PIK3R1) and Never in Mitosis gene a-(NIMA-)Related Kinase 2 (NEK2), which have been shown to play important roles in HCC proliferation, migration, and invasion [23,24]. Furthermore, miRNA-486-5p is located on chromosome 8q11.21, an area commonly deleted in HCC, which possibly accounts for reduced tissue expression in HCC and supports its potential role as a tumour suppressor [25]. Our finding of differential serum expression of miRNA-486-5p in HCC further promotes the importance and clinical potential of this miRNA.

The current study demonstrated significantly reduced expression of circulating miRNA-151a-5p in cirrhotic patients with HCC compared with cirrhosis alone. Others have described increased plasma expression of miRNA-151a-5p in viral hepatitis-induced HCC, but this was in comparison with healthy controls and non-viral hepatitis-related HCC patients [26]. Indeed, increased tissue expression levels of miRNA-151a-5p have been described in HCC tissue, compared with adjacent non-tumorous tissue [25,27], and miRNA-151a-5p is located on chromosome 8q24.3, which is an area that is frequently amplified in HCC [25,27]. High tissue expression levels of miRNA-151a-5p have been correlated with intrahepatic metastasis, cell migration, and invasion [25], while *FAK* (the focal adhesion kinase gene that is the host gene of miRNA-151a-5p) is usually co-expressed with miRNA-151a-5p, and it has been suggested that the two function synergistically to enhance HCC cell motility [25]. There is, therefore, growing evidence that miRNA-151a-5p may have a pro-oncogenic function, highlighting the clinical potential of identifying it as one of the differentially-expressed candidates in our cohort.

We performed ROC curve analysis using stepwise logistic regression to identify an optimal miRNA panel to distinguish cirrhosis from mild disease in CHC. The resulting cirrhosis miRNA panel (comprising miRNA-122-5p and miRNA-409-3p) resulted in an AUC of 0.80. Combining the panel with APRI or FIB-4 only marginally improved the performance of either score alone (AUCs of 0.87 and 0.89, respectively), but illustrates the future clinical potential of combining miRNA expression with other non-invasive biomarkers to improve diagnostic accuracy. ROC curve analysis of our optimal HCC miRNA panel, comprising miRNA-122-5p, miRNA-486-5p, and miRNA-142-3p, demonstrated an excellent ability to distinguish patients with HCC from those with cirrhosis alone, with an AUC of 0.94. This result was substantially better than the diagnostic performance of AFP to identify HCC (AUC of 0.64). It also statistically out-performed the more intuitive combination of miRNA-122-5p, miRNA-486-5p, and miRNA-151a-5p (AUC 0.91) due to co-linear expression of miRNA-122-5p and miRNA-151a-5p. Combining AFP with our miRNA panel did not improve its diagnostic performance. While prospective validation of our HCC miRNA panel in a larger cohort is warranted, these results clearly demonstrate the clinical potential of this serum miRNA panel, with improved sensitivity (80% vs. 25%) and specificity (95% vs. 90%) compared with the only HCC tumour biomarker routinely used in clinical practice (AFP).

This study contributes to the considerable growing interest in miRNAs as biomarkers of chronic liver disease in recent years. Meta-analyses [28,29] have concluded that miRNAs have great potential as non-invasive biomarkers, especially in the diagnosis of HCC, and a number of potential miRNAs candidates have been identified. These analyses found, however, that the quality of studies in this area is highly variable, significant heterogeneity exists, and results are often contradictory. Indeed, the published data suggest that high frequency miRNAs (such as miRNA-122-5p in HCC) may ultimately be the most specific candidates, and combinations of non-invasive biomarkers and/or panels of miRNAs are now believed to be the most likely strategy in accurately distinguishing disease [29]. Our data are consistent with these conclusions. We identified that optimal diagnostic performance was achieved using combinations of significantly differentially-expressed miRNAs, including commonly-expressed miRNAs. Further high-quality prospective research is needed to validate these promising preliminary results. The current study also confirmed a number of candidate miRNAs that have been implicated in other HCC studies, including a novel approach that assessed circulating miRNA levels significantly associated with HCC in viral hepatitis in an attempt to establish a predictive model [30]. Such candidates could be further investigated in functional studies to identify downstream targets to elucidate their roles in disease pathogenesis and potentially contribute to the development of new therapeutic strategies.

There are some limitations to this study that need to be considered when evaluating our results. First, our study population only included two patients who had achieved an SVR and genotypes 1 and 3 were also over-represented in our cohort, as they are the most common genotypes in Australia. A clinically useful diagnostic miRNA panel would ideally demonstrate accuracy across all genotypes and also in cirrhotic patients following viral eradication; second, in the HCC cohort, serum samples were not obtained following definitive HCC treatment meaning dynamic changes of our proposed diagnostic serum miRNAs according to tumour viability could not be assessed. While this could lend additional weight to our results, this was not undertaken in the current study as treatments (and therefore outcomes) varied considerably amongst the cohort, thus making data difficult to interpret. Additionally, there was the risk of a type 2 error in the interpretation of any data due to the sample size in this pilot cohort; third, patients in this study were allocated to clinically-significant cohorts based on standard-of-care expert assessment instead of being formally staged with gold standard diagnostic biopsy, meaning discriminating between individual fibrosis stages based on miRNA expression was beyond the scope of this study. Clearly, a study using both current standard-of-care clinical assessment and liver biopsy would be desirable, but treatment of Hepatitis C in the era of highly efficacious direct-acting antivirals rarely involves liver biopsy; finally, and perhaps most importantly, this was a pilot study with small numbers of (albeit well-characterised) patients that aimed to test hypotheses, generate preliminary data, and contribute to our understanding of this area. To address the robustness of our data, we performed an internal 5-fold validation, but we acknowledge the importance of an external, preferably prospective, validation study from investigators with sufficiently large patient cohorts.

Compared with recent studies, however, there are a number of important methodological strengths of the current study. First, we used clinically-relevant controls (i.e., mild disease that is definitely not cirrhosis vs. established cirrhosis; cirrhosis alone vs. cirrhosis complicated by HCC) instead of healthy controls. Additionally, as even gold standard liver biopsy is associated with fibrosis stage sampling errors, we suggest that the use of these clinically-relevant patient cohorts, rather than sub-dividing patients according to individual *METAVIR* stages, may provide more meaningful data to investigators; second, we measured circulating rather than tissue-based miRNA levels as peripheral samples are much more accessible to clinicians and a blood-based diagnostic biomarker has huge logistic advantages over a tissue-based one; finally, we compared expression levels in serum rather than plasma, which, as is increasingly accepted, deliver suboptimal results [29]. We suggest that the results of this pilot study, taken in the context of emerging data in this field, represent an important contribution to the literature and further highlight the roles of specific miRNAs as promising non-invasive biomarkers. The excellent performance of our miRNA panel, compared with AFP as the only clinically-accepted biomarker in HCC, also suggests that we may not be too far away from a validated diagnostic HCC miRNA panel that could be used in clinical practice.

## 4. Materials and Methods

### 4.1. Patient Recruitment and Characteristics

All study procedures were undertaken in accordance with organisational ethical standards on human experimentation with approval of the Human Research Ethics Committees of the Royal Brisbane and Women’s Hospital (HREC/13/QRBW/308, approval 12/12/2013) and QIMR Berghofer Medical Research Institute (P1509, approval 07/12/2012), and in accordance with the Declaration of Helsinki 1975 (revised 2013). Informed consent was obtained from all patients. Sixty CHC patients were retrospectively subdivided into 3 cohorts as follows: Mild disease without advanced fibrosis (F0-2; *n* = 20); cirrhosis (F4; *n* = 20); and cirrhosis with HCC (HCC; *n* = 20). Detailed patient characteristics are described in Supplementary Methods and Supplementary Table S1.

### 4.2. RNA Extractions and Reverse Transcription

For the screening phase, RNA was extracted from serum using the miRNeasy Serum/Plasma Kit (Qiagen; Hilden, Germany) and reverse-transcribed using the miScript II RT Kit (Qiagen). During the validation phase, serum RNA was extracted using the Plasma/Serum RNA Purification Mini Kit (Norgen Biotek Corp; Thorold, ON, Canada) and reversed-transcribed using the miRCURY LNA^TM^ universal RT miRNA PCR Kit (Exiqon, Vedbaek, Denmark). RNA extraction, protocol modifications, and reverse transcription are described in Supplementary Methods. Primer target sequences for candidate miRNAs are shown in Supplementary Table S2.

### 4.3. miRNA PCR Array, qRT-PCR, and Data Analysis

During the screening phase, a miRNA PCR Array (Human Liver miFinder miScript miRNA PCR Array MIHS-3116ZG; Qiagen) was used to simultaneously measure the expression of 372 liver-related miRNAs in all 60 samples. The most significant differentially-expressed miRNAs (>2-fold change and *p* < 0.05) were selected for further validation. Leading miRNA candidates were independently validated by qRT-PCR (miRCURY LNA™ miRNA kit, see Supplementary Methods).

Following the screening phase, miRNA array data were normalised using quantile normalisation and analysed for differentially-expressed miRNAs using pairwise comparison of groups of interest with the modified t-test (limma package, R). miRNA candidates were selected based on *p* < 0.05. Validation qRT-PCR data were analysed using the 2^Δ*C*t^ method and expression values normalized to let-7i-5p and miRNA-23a-3p. Significant differential expression was tested using one-way ANOVA with Tukey’s post-hoc pairwise comparison, with *p* < 0.05 used to define statistical significance. Data normalisation and statistical analysis were performed using GraphPad Prism 7 (GraphPad Software Inc; San Diego, CA, USA) and R (version 3.3.3, https://www.r-project.org) [31].

### 4.4. Panel Design and k-Fold Cross Validation

Stepwise logistic regression using forward selection and backward elimination was used to derive miRNA panels for (i) cirrhosis (F4) vs. mild disease (F0-2), and (ii) HCC vs. cirrhosis (F4). Pairwise correlations between individual miRNAs were performed to exclude miRNA combinations that displayed significant co-linearity within the model. K-fold cross-validation (5-fold) was used to assess the performance of the selected miRNA panels. The detailed panel design and k-fold cross validation methodology are described in Supplementary Methods.

## 5. Conclusions

This study identified the differential expression of several circulating miRNAs across a clinically-relevant spectrum of disease severity due to CHC. Combining the expression levels of different miRNAs allows the optimisation of diagnostic panels that could potentially be developed to stratify CHC and diagnose early HCC. Prospective external validation of our miRNA panels is required, but these exciting preliminary results support the potential use of miRNAs as non-invasive biomarkers in the clinical assessment of CHC patients. We also provide further evidence that a miRNA panel could ultimately replace AFP in clinical practice as the non-invasive biomarker of choice in HCC.

## 6. Patents

A Complete Patent application (Australia and US only) was filed, entitled ‘DETECTION OF LIVER DISEASE’ (AU2018202716; Inventors Anna Weis, Richard Skoien, Grant Ramm).

## Figures and Tables

**Table 1:** Associations of circulating miRNA expression with the severity of disease in chronic Hepatitis C: Validation of selected miRNA expression levels using qRT-PCR.

miRNA	Overall *p*-Value (ANOVA)	*p*-Value		
		F0-2 vs. F4	F0-2 vs. HCC	F4 vs. HCC
miRNA-122-5p	<0.001	0.066	0.103	<0.001
miRNA-486-5p	<0.001	0.999	0.001	0.001
miRNA-151a-5p	0.025	0.986	0.056	0.039
miRNA-409-3p	0.030	0.023	0.417	0.320
miRNA-19b-3p	0.015	0.734	0.015	0.089
miRNA-142-3p	0.047	0.636	0.039	0.253

*p*-values were calculated using the 2^Δ*C*t^ normalisation method and ANOVA with Tukey’s post hoc test with significance designated by a *p*-value < 0.05. (Abbreviations: ANOVA = analysis of variance; F0-2 = mild disease; F4 = cirrhosis; HCC = cirrhosis with hepatocellular carcinoma; miRNA = microRNA; qRT-PCR = quantitative real time reverse transcription PCR).

## Supplementary Materials

Supplementary Materials can be found at http://www.mdpi.com/1422-0067/20/4/864/s1.

## Abbreviations

AFP	Alpha-fetoprotein
ALT	Alanine aminotransferase
APRI	Aspartate aminotransferase to platelet ratio
AST	Aspartate aminotransferase
AUC	Area under the curve
CHC	Chronic Hepatitis C
95% CI	95% Confidence interval
DAA	Direct acting antivirals
FAK	Focal adhesion kinase
FIB-4	Fibrosis-4
HCC	Hepatocellular carcinoma
IQR	Interquartile range
miRNA	MicroRNA
NEK2	NIMA-related kinase 2
NIMA	Never in mitosis gene a
NPV	Negative predictive value
PIK3R1	Phosphoinositide-3-Kinase Regulatory Subunit 1
PPV	Positive predictive value
qRT-PCR	Real-time quantitative reverse transcription PCR
ROC	Receiver operating characteristic
SVR	Sustained virologic response
