# Supplementary material for: Serum MicroRNAs as Biomarkers in Hepatitis C: Preliminary Evidence of a MicroRNA Panel for the Diagnosis of Hepatocellular Carcinoma
Source: Int J Mol Sci. 2019 Feb 17;20(4):864. doi: 10.3390/ijms20040864 (PMC6412219; doi:10.3390/ijms20040864)
Supplement: Supplementary file 1 [file ijms-20-00864-s001.pdf]

International Journal of *Molecular Sciences*

MDPI

Article

Serum MicroRNAs as Biomarkers in Hepatitis C: Preliminary Evidence of a MicroRNA Panel for the Diagnosis of Hepatocellular Carcinoma

7. Supplementary Results

7.1. Patient characteristics.

The patient demographics and clinical characteristics of the CHC patient cohort are summarised in Supplementary Table S1. Paired serum samples for miRNA testing and routine laboratory assessment of disease status were obtained with 95% of patients' samples taken within 4 months of each other. As expected, traditional markers of disease severity (*e.g.*, ALT, AST, sodium and platelet levels) were significantly different amongst the three study groups (all $P<0.001$), whereas creatinine levels were similar (Supplementary Table S1). Markers of hepatic function (*i.e.*, bilirubin, albumin, INR) were significantly worse in patients with HCC compared with those with cirrhosis alone (all $P<0.001$). Interestingly, while the tumour marker AFP was increased in HCC vs. cirrhosis alone, this difference was not statistically significant ($P=0.129$) due to the wide variation of AFP values in the HCC cohort (Supplementary Table S1).

7.2. Discriminating cirrhosis and HCC using serum miRNA panels.

The utility of either serum miRNA-409-3p and miRNA-122-5p to distinguish cirrhosis from mild disease was fair with AUCs of 0.74 ($P=0.004$) and AUC of 0.69 ($P=0.023$), respectively. The individual performance of several serum miRNA candidates to distinguish HCC patients from those with cirrhosis alone was better with AUCs of 0.85 ($P<0.001$) for miRNA-122-5p and 0.78 ($P<0.001$) for miRNA-486-5p, while the utility of miRNA-151a-5p (AUC=0.70, $P=0.014$) and miR142-3p (AUC=0.60, $P=0.14$) was fair (Figure 2).

8. Supplementary Materials and Methods

8.1. Patient recruitment and characteristics

Sixty HCV-positive patients were retrospectively subdivided into 3 cohorts based on expert clinical assessment, transient elastography (FibroScan™; Echosens, Paris, France) and medical imaging as follows: mild disease without advanced fibrosis (F0-2; n=20); cirrhosis (F4; n=20); and cirrhosis with HCC (HCC; n=20). The diagnosis of HCC was established according to currently-accepted professional guidelines.[1] Patient demographics, biochemistry and relevant medical information were obtained from patients' medical records. Serum samples were obtained through Pathology Queensland collection centres. All samples were processed within 8 hours of blood draw and stored at -80°C. A serum sample was collected from each patient and corresponding APRI (applying a cut-off of 1.0 to exclude cirrhosis),[2] FIB-4 (using a cut-off of 1.45 to exclude advanced fibrosis),[3] Child-Turcotte-Pugh (CTP) and model for end-stage liver disease (MELD) scores were calculated.

8.2. RNA extractions and reverse transcription.

For the screening phase, RNA was extracted from 200 µl of serum using the miRNeasy Serum/Plasma Kit (Qiagen; Hilden, Germany). Isolations were performed according to

manufacturer's instructions with minor modifications to optimise results. Following the phenol-chloroform phase separation, the interphase and organic layers were rehydrated with RNAse free water equal to the volume removed during the aqueous phase collection.[4] Thus, a second aqueous phase was obtained maximizing RNA recovery. Both aqueous phases were combined into one RNeasy MiniElute spin column and RNA isolation was continued.[4] Following RNA elution, a second elution with identical settings was performed using 14µl RNAse free water in a new collection tube. All samples were assessed for ethanol contamination and RNA yield by Nanodrop™ (Thermo Fisher Scientific; Waltham, MA, USA). Serum extracted RNA was reverse transcribed using the miScript II RT Kit (Qiagen) following the manufacturer's instructions and RNA input recommendation for miScript miRNA PCR Arrays with 250 ng RNA. cDNA products were diluted 10-fold prior to microRNA PCR array run. During the validation phase, serum RNA was extracted using the Plasma/Serum RNA Purification Mini Kit (Norgen Biotek Corp; Thorold, Ontario, Canada) following the manufacturer's instructions with minor modifications. Prior to transferring the sample onto the Micro Spin Column the mixture was passed through a syringe with a 26G needle to break down lysis debris and prevent column clogging. RNA was eluted in 15 µl of RNAse free water and assessed on a Nanodrop™. Extracted RNA was reversed transcribed using the miRCURY LNA™ universal RT microRNA PCR Kit (Exiqon, Vedbaek, Denmark) following manufacturer's instructions. A fixed RNA volume input of 4 µL was used (at the manufacturer's suggestion) due to limitations in quantifying circulatory microRNAs. cDNA products were diluted 1:40 prior to qRT-PCR use.

8.3. MiRNA PCR Array, qRT-PCR and data analysis.

During the screening phase, a miRNA PCR Array (Human Liver miFinder miScript miRNA PCR Array MIHS-3116ZG; Qiagen) was used to simultaneously measure expression of 372 liver-related miRNAs in all 60 samples. The most significant differentially-expressed miRNAs, (>2-fold change and P<0.05) were selected for further validation. Leading miRNA candidates were independently validated by qRT-PCR (miRCURY LNA™ miRNA kit and Exiqon primers, Supplementary Table 2). The miRNA PCR Array and miRCURY miRNA qRT-PCR were performed using the Lightcylcer480 (Roche; Basel, Switzerland) and CFX384 (Bio-Rad; Hercules, California) thermal cycler, respectively. MiRNA PCR Array and miRCURY qRT-PCR data were reported as crossing points (CP) and quantification cycles (Cq), respectively.

Statistical analysis of patient demographics was undertaken using the chi-square test for categorical variables, and either an ANOVA, Kruskal-Wallis test, unpaired t-test or Mann-Whitney test for continuous variables as informed by the D'Agostino & Pearson normality test. Significance was defined as P<0.05.

The endogenous reference miRNAs let-7i-5p and miRNA-23a-3p were selected for further validation studies based on a distance metric ranking combining the P-values from the pairwise comparisons of comparing mild disease (F0-2) vs cirrhosis (F4) and comparing cirrhosis (F4) vs HCC. The distance metric was calculated as the summation of the absolute value of the log transformed P-values. Validation qRT-PCR data were analysed using the $2^{\Delta CT}$ method and expression values normalized to let-7i-5p and miRNA-23a-3p.

8.4. Panel design and k-fold cross validation.

Stepwise logistic regression using forward selection and backward elimination was used to derive microRNA panels for i) cirrhosis (F4) vs mild disease (F0-2), and ii) HCC vs cirrhosis (F4). Pairwise correlations between each of the miRNA were assessed to exclude any significant correlations within the models at the 5% level of significance. Model selection was based on the

Akaike Information Criteria, the likelihood ratio test based on the change of the residual deviance, and by assessing the stability of the coefficient estimates.

The resulting model equation is as follows:

Function A: F4 vs F0-2

$$\ln(\frac{p_i}{1-p_i}) = 4.03 - (0.89 \times \Delta\text{CT miR-122-5p}) - (0.83 \times \Delta\text{CT miR-409-3p})$$

Function B: HCC vs F4

$$\ln(\frac{p_i}{1-p_i}) = 3.59 + (1.78 \times \Delta\text{CT miR-122-5p}) - (1.99 \times \Delta\text{CT miR-486-5p}) + (2.32 \times \Delta\text{CT miR-142-3p})$$

To obtain the p_i , the probability of the ith subject having the outcome of cirrhosis (function A) or HCC (function B), use the following formula where *function* is the right hand side of the equation in Function A or B:

$$p_i = \frac{1}{1 + \exp\,(-function)}$$

The probability, p_i, will range from 0 to 1.

The Youden's index is a technique used to determine the most appropriate cut-off value, which corresponds to a point on the ROC curve with the highest vertical distance from the 45% diagonal line. At this point, the true positive rate and the false positive rate is at the maximum possible.[5] Using the Youden's index a panel specific cut-point for p_i was determined. For the probability p_i derived using function A, a value above the cut-point of 0.44 would classify the subject as cirrhosis. For the probability p_i derived using function B, a value above the cut-point of 0.65 would classify the subject as HCC.

K-fold cross-validation (5-fold) was used to assess the performance of the selected microRNA panels. Univariate and multivariable analyses were used to perform receiver operating characteristic (ROC) curve analysis resulting in area under the curve (AUC) with 95% confidence interval (CI), positive predictive value (PPV), negative predictive value (NPV), sensitivity, specificity and accuracy. All calculations were performed using the cvAUC and the ROCR package on R version (version 3.3.3).[6]

Supplementary Tables

Supplementary Table S1. Demographic and clinical characteristics of patients across all study groups

	**Mild fibrosis (n=20)**	**Cirrhosis (n=20)**	**HCC (n=20)**	**P-value**
Gender[1]				0.045
Male (n/%)	10 (50%)	17 (85%)	15 (75%)	
Female (n/%)	10 (50%)	3 (15%)	5 (25%)	
Age (years)[2]	49.6 (SD 10.1)	53.8 (SD 7.4)	58.3 (SD 5.3)	0.004
BMI[2]	26.3 (SD 6.0)	27.2 (SD 5.1)	27.1 (SD 5.2)	0.864
HCV genotype (n)[1]				0.695
1	1 (5%)	-	2 (10%)	
1a	8 (40%)	8 (40%)	6 (30%)	
1b	3 (15%)	4 (20%)	2 (10%)	

Mixed 1/3	1 (5%)	-	-	
2	1 (5%)	-	-	
3	6 (30%)	6 (30%)	9 (45%)	
4	NA	1 (5%)	NA	
Prior SVR	NA	1 (5%)	1 (5%)	
HCV treatment history[1]				0.065
Treatment experienced	6 (30%)	6 (30%)	6 (30%)	
Treatment naïve	14 (70%)	14 (70%)	10 (50%)	
On DAA treatment	-	-	4 (20%)	
Baseline routine laboratory tests				
Sodium (mmol/L)[2]	138.5 (SD 2.7)	137.6 (SD 1.4)	135.9 (SD 2.4)	0.002
creatinine (μmol/L)[2]	68.2 (SD 14.6)	71.3 (SD 13.4)	68.2 (SD 16.9)	0.761
ALT (U/L)[3,6]	63.5 (IQR 46.0-97.3)	155.5 (IQR 105.5-223.5)	55 (IQR 34.3-88.5)	<0.001
AST (U/L)[3,6]	39.5 (IQR 30.0-70.3)	123.5 (IQR 78.5-235.0)	75.5 (IQR 53.8-101.3)	<0.001
Platelet ($x10^{9}$/L)[2]	226.3 (SD 57.6)	164.4 (SD 32.8)	124.7 (SD 63.2)	<0.001
bilirubin (μmol/L)[4]	NA	14.5 (SD 4.5)	25.3 (SD 10.0)	<0.001
albumin (g/L)[4]	NA	40.4 (SD 2.8)	33.7 (SD 6.1)	<0.001
Proximity of sample collection to baseline routine laboratory tests (months)	1.47 (SD 6.40)	0.22 (SD 1.45)	0.01 (SD 0.76)	NA
Other laboratory tests				
INR4	NA	1.1 (SD 0.1)	1.2 (SD 0.1)	<0.001
AFP (μg/L)[5,6]	NA	5.8 (IQR 3.3-13.0)	8.6 (IQR 8.0-21.3)	0.129
Sample collection to AFP (months)	NA	0.03 (SD 2.48)	0.48 (SD 1.55)	NA
Clinical follow up (months)	16.7 (SD 7.2)	21.2 (SD 6.7)	3.3 (SD 3.0)	NA

	Mild fibrosis (n=20)	Cirrhosis (n=20)	HCC (n=20)	P-value
Disease severity assessment Transient elastography LSM (kPa)[4]	6.1 (SD 1.2)	19.2 (SD 8.5)	NA	<0.001
FIB-4[5,6]	1.2 (IQR 0.9-1.9)	3.2 (IQR 2.7-4.6)	NA	<0.001
APRI[5,6]	0.6 (IQR 0.3-1.2)	2.3 (IQR 1.1-4.5)	NA	<0.001
CTP				NA

A	NA	20 (100%)	8 (40%)	
B	NA	-	11 (55%)	
C	NA	-	1 (5%)	
MELD	NA	7.31 (SD 0.67)	10.26 (SD 2.38)	NA
BCLC (n/%)				NA
0	NA	NA	2 (10%)	
A	NA	NA	8 (40%)	
B	NA	NA	8 (40%)	
C	NA	NA	1 (5%)	
D	NA	NA	1 (5%)	

Following normality testing, P-values were calculated using the [1]Chi-Square test, [2]ANOVA, [3]Kruskal-Wallis test, [4]unpaired t-test and [5]Mann-Whitney test with significance defined as a P-value < 0.05. [6]Data shown as median. Data are shown as mean values unless otherwise specified. (Abbreviations: AFP, alpha-fetoprotein; ALT, alanine aminotransferase; APRI, aspartate aminotransferase to platelet ratio; AST, aspartate aminotransferase; BCLC, Barcelona clinic liver cancer; BMI, body mass index; CTP, Child-Turcotte-Pugh Score; DAA, direct acting antiviral drug; Fib-4; Fibrosis-4; HCC, hepatocellular carcinoma; HCV, Hepatitis C virus; INR, international normalized ratio; IQR, interquartile range; LSM, liver stiffness measurement MELD, model for end-stage liver disease score; NA, not applicable; SD, standard deviation; SVR, sustained virologic response).

Supplementary Table S2: Target qRT-PCR primer sequences for candidate miRNAs during the validation phase.

No.	microRNA name	Description	Target Sequence	Product No. Exiqon
1	miR-19b-3p	hsa-miR-19b-3p LNA™ PCR primer set, UniRT	UGUGCAAAUCCAUGCAAAACUGA	204450
2	miR-151a-5p	hsa-miR-151a-5p LNA™ PCR primer set, UniRT	UCGAGGAGCUCACAGUCUAGU	204007
3	miR-122-5p	hsa-miR-122-5p LNA™ PCR primer set, UniRT	UGGAGUGUGACAAUGGUGUUUG	205664
4	miR-409-3p	hsa-miR-409-3p LNA™ PCR primer set, UniRT	GAAUGUUGCUCGGUGAACCCCU	204358
5	miR-22-3p	hsa-miR-22-3p LNA™ PCR primer set, UniRT	AAGCUGCCAGUUGAAGAACUGU	204606
6	miR-142-3p	hsa-miR-142-3p LNA™ PCR primer set, UniRT	UGUAGUGUUUCCUACUUUAUGGA	204291
7	miR-486-5p	hsa-miR-486-5p LNA™ PCR primer set, UniRT	UCCUGUACUGAGCUGCCCCGAG	204001
8	let-7i-5p	hsa-let-7i-5p LNA™ PCR primer set, UniRT	UGAGGUAGUAGUUUGUGCUGUU	204394
9	miR-23a-3p	hsa-miR-23a-3p LNA™ PCR primer set, UniRT	AUCACAUUGCCAGGGAUUUCC	204772

References

1. [1] J. Bruix, M. Sherman, and D. American Association for the Study of Liver, "Management of hepatocellular carcinoma: an update," *Hepatology,* vol. 53, pp. 1020-2, Mar 2011.

2. [2] Z. H. Lin, Y. N. Xin, Q. J. Dong, Q. Wang, X. J. Jiang, S. H. Zhan, *et al.*, "Performance of the aspartate aminotransferase-to-platelet ratio index for the staging of hepatitis C-related fibrosis: an updated meta-analysis," *Hepatology,* vol. 53, pp. 726-36, Mar 2011.
3. [3] R. K. Sterling, E. Lissen, N. Clumeck, R. Sola, M. C. Correa, J. Montaner, *et al.*, "Development of a simple noninvasive index to predict significant fibrosis in patients with HIV/HCV coinfection," *Hepatology,* vol. 43, pp. 1317-25, Jun 2006.
4. [4] K. L. Burgos, A. Javaherian, R. Bomprezzi, L. Ghaffari, S. Rhodes, A. Courtright, *et al.*, "Identification of extracellular miRNA in human cerebrospinal fluid by next-generation sequencing," *RNA,* vol. 19, pp. 712-22, May 2013.
5. [5] F. Habibzadeh, P. Habibzadeh, and M. Yadollahie, "On determining the most appropriate test cut-off value: the case of tests with continuous results," *Biochem Med (Zagreb),* vol. 26, pp. 297-307, Oct 15 2016.
6. [6] R. C. Team, "R: A language and environment for statistical computing. R Foundation for Statistical Computing, Vienna, Austria. 2013," ed: ISBN 3-900051-07-0, 2014.

© 2019 by the authors. Submitted for possible open access publication under the terms and conditions of the Creative Commons Attribution (CC BY) license (http://creativecommons.org/licenses/by/4.0/).
